# Onlay Repair Technique for the Management of Ureteral Strictures: A Comprehensive Review

**DOI:** 10.1155/2020/6178286

**Published:** 2020-07-27

**Authors:** Shengwei Xiong, Jie Wang, Weijie Zhu, Kunlin Yang, Guangpu Ding, Xuesong Li, Daniel D. Eun

**Affiliations:** ^1^Department of Urology, Peking University First Hospital, No. 8 Xishiku St, Xicheng District, Beijing 100034, China; ^2^Institute of Urology, Peking University, No. 8 Xishiku St, Xicheng District, Beijing 100034, China; ^3^National Urological Cancer Center, No. 8 Xishiku St, Xicheng District, Beijing 100034, China; ^4^Department of Urology, Temple University School of Medicine, 255S 17th Street, 7th Floor Medical Tower, Philadelphia, PA 19103, USA

## Abstract

Ureteroplasty using onlay grafts or flaps emerged as an innovative procedure for the management of proximal and midureteral strictures. Autologous grafts or flaps used commonly in ureteroplasty include the oral mucosae, bladder mucosae, ileal mucosae, and appendiceal mucosae. Oral mucosa grafts, especially buccal mucosa grafts (BMGs), have gained wide acceptance as a graft choice for ureteroplasty. The reported length of BMG ureteroplasty ranged from 1.5 to 11 cm with success rates of 71.4%-100%. However, several studies have demonstrated that ureteroplasty using lingual mucosa grafts yields better recipient site outcomes and fewer donor site complications than that using BMGs. In addition, there is no essential difference in the efficacy and complication rates of BMG ureteroplasty using an anterior approach or a posterior approach. Intestinal graft or flap ureteroplasty was also reported. And the reported length of ileal or appendiceal flap ureteroplasty ranged from 1 to 8 cm with success rates of 75%-100%. Moreover, the bladder mucosa, renal pelvis wall, and penile/preputial skin have also been reported to be used for ureteroplasty and have achieved satisfactory outcomes, but each graft or flap has unique advantages and potential problems. Tissue engineering-based ureteroplasty through the implantation of patched scaffolds, such as the small intestine submucosa, with or without cell seeding, has induced successful ureteral regeneration structurally close to that of the native ureter and has resulted in good functional outcomes in animal models.

## 1. Introduction

A ureteral stricture is characterized by a narrowing of the ureter that causes a functional obstruction. As urinary drainage becomes restrained, the urine is stagnated in the upper tract and renal pelvis. This condition may cause renal pain and may lead to urinary tract infections or even renal failure if left untreated [1–3]. Ureteral strictures can be classified into proximal, middle, and distal ureteral strictures; panureteral strictures; and ureteropelvic junction obstruction (UPJO), according to the sites of stricture [4]. Short-segment strictures of the proximal and middle ureter are usually reconstructed by endourological management or surgical operations involving primary excision and end-to-end anastomosis either in the form of pyeloureteroplasty or ureteroureterostomy [5, 6]. If a longer segment stricture is present, more advanced surgical techniques, such as renal mobilization and downward nephropexy, ileal ureter replacement, transureteroureterostomy, and autotransplantation of the kidney, are necessary to provide a tension-free anastomosis. Unlike a proximal stricture, a distal ureteral stricture is usually managed with ureteral reimplantation, achieving additional length with a downward nephropexy procedure, a psoas hitch, or a Boari flap technique [7].

However, kidney autotransplantation requires high expertise during transplant surgery and may cause susceptibility to significant renovascular morbidity [8]. In addition, the incorporation of a long bowel segment into the urinary tract is associated with severe metabolic and intestinal complications [9]. These issues pose a difficult management dilemma for proximal and middle stricture, and alternative options are urgently needed. Although the strictured ureter is insufficient to achieve completely patent drainage, it can still provide a “ureteral plate” with minimally destructed blood supply after excising partial circumference. Based on this theory, an onlay repair technique emerged. Recently, onlay ureteroplasty with grafts or flaps has been attempted by many reconstructive urologists and has yielded encouraging outcomes. Herein, we mainly evaluate the efficacy of some autologous materials and tissue-engineered material for onlay ureteroplasty in the management of ureteral strictures and present an updated review of this innovative technique.

## 2. Histologic Considerations of Tissue Transferring

A unique aspect of a graft in ureteral reconstruction is that the tissue is excised from the donor site and transferred to the recipient site where a new blood supply develops [10]. A flap is vascularized tissue that is transferred to the recipient site, maintaining its own blood supply [11]. The selection of the tissues for ureteroplasty is based on the characteristics of the ureteral strictures and the patient's global situation. The morbidity of the donor site and the function of the site to be reconstructed should also be considered. The success of tissue transfer technique is dependent on a good “take” process through the rapid onset of the plasmatic imbibition and inosculation phases, which are optimized with well-vascularized recipient beds, good apposition, and the immobilization of the grafts [11]. In addition, grafts or flaps with a thick epithelium, a thin lamina propria, and an abundant capillary plexus accelerate the imbibition and inosculation phases [12]. Graft failure is usually caused by fluid accumulation, such as hematoma or seroma, under the graft. This situation can be prevented through creating perforations in the graft, graft meshing, and bolster dressing or vacuum-assisted closure [13].

To be suitable for incorporation into the urinary tract, a graft should be hairless, easy to access and harvest, and viable in a urinary environment. A number of different tissues that can be categorized as either mucosal grafts or skin grafts have been used for the purpose of ureteral reconstruction. After long-term exposure to urine, some mucosal grafts retained their histopathological characteristics and showed no significant inflammatory cell infiltration or erosion in animal models [14] and human studies [15]. In contrast, there were a severe inflammatory reaction, hyalinization, erosion, and shrinkage of the full-skin grafts [14]. The commonly used autologous mucosal tissues include the oral mucosae (buccal and lingual mucosae), bladder mucosae, ileal mucosae, and appendiceal flaps.

## 3. OMG Ureteroplasty

### 3.1. Buccal Mucosa Graft

The oral mucosa is hairless, easily accessible, easy to harvest, and compatible with a wet environment. The common sites of oral mucosa graft (OMG) harvesting include the inner cheek or lip (buccal mucosa) and the lateral or ventral surface of the tongue (lingual mucosae). The buccal and lingual mucosae have the same tissue characteristics, including a thick epithelium, high content of elastic fibers, thin lamina propria, and high capillary density, which are beneficial for promoting revascularization. Using buccal mucosa as a tube or an onlay/inlay graft for the treatment of complex ureteral strictures has been reported previously. Onlay repair means a graft applied or laid on the surface of a structure, while inlay repair means a graft inlaid or inserted in the cavity of a structure [16]. And tubularized repair means a graft reconfigured in tubular form. Currently, buccal mucosa graft (BMG) has gained wide acceptance as a graft choice for onlay ureteroplasty. The pioneering attempt at using BMG as a nonpedicled, full-thickness tubularized graft for ureteral reconstruction was implemented on three baboons by Somerville and Naude in 1983 [17]. Encouraged by the results of buccal mucosal ureteral replacement in animal studies and the recognized new gold standard of BMG urethroplasty for the repair of urethral strictures or hypospadias [18], the initial experience with human ureteroplasty with BMG was reported by Naude in 1999 [19]. Five patients who had complicated ureteral strictures caused by various diseases were treated with buccal mucosa patch grafts and an omental wrap. In all patients, the grafts maintained good patency and drainage, and there were no stricture recurrences after long-term follow-up [19].

Till now, many single case reports [20–23] and case series reports (as shown in Table 1) described the success of BMG onlay ureteroplasty. The follow-up was from 3 to 85 months with success rates of 71.4%-100%. The length of ureteral repair ranged from 1.5 cm to 11 cm. Kroepfl et al. reported the reconstruction of long ureteral strictures utilizing buccal mucosal patch grafts and omental wrapping in six patients [24]. And they described the longest length (11 cm) of ureteral stricture reconstructed by BMG ureteroplasty. All patients showed good functional outcomes at an intermediate-term follow-up. Recurrent strictures below the reconstructed ureter segment, causing impaired urinary drainage, were found in both the patients with the longest segment BMG ureteroplasty and those with bilateral BMG ureteroplasty. However, how the reconstructed length and position of the ureter influence urinary drainage remains speculative. The authors assumed that this situation most likely resulted from the misjudgment of the distal extent of the original stricture [24]. The initial incorporation of the robotic-assisted technique into BMG ureteroplasty was reported by Zhao et al. for the proximal or multifocal ureteral reconstruction of four patients [25]. At a median follow-up of 15.5 months, all patients demonstrated no hydronephrosis, and the patency of the reconstructed ureter was confirmed. In this report, the patched grafts were placed in the ureters in an anterior or posterior fashion, and one patient received an augmented anastomotic procedure, which required excision of the diseased segment and posterior anastomosis of the two ends of the healthy ureter to create the position for the anterior onlay. In patients with anterior BMG onlays, an omentum was immobilized in place after the suture of BMG was complete. In patients with posterior BMG patches, an omentum was sutured in place ahead of the ureterotomy [25]. It is debatable whether posterior BMG patching has advantages over the anterior approach. Dorsal patching may reduce the possibility of diverticulum formation of the graft, but ventral patching is technically easier to perform as the ureter does not need to be rotated. This rotation may be difficult to perform when the ureteral stricture is close to the renal hilum. However, there is no essential difference in the efficacy and complication rates of using an anterior approach or a posterior approach at long-term follow-up [26].

More recently, Zhao et al. reported a multi-institutional experience of 19 patients treated with robotic BMG ureteroplasty, representing the largest reported series to date [26]. The onlay ureteroplasty procedure was carried out in 79% of patients, and the augmented anastomotic technique was used for the remaining patients. At a median follow-up of 26 months, 90% of patients had successful clinical and radiological outcomes. In addition, the authors attached extra importance to the utilization of intraoperative flexible ureteroscopy with near-infrared fluorescence (NIRF) imaging to assist with the precise identification of the proximal and distal margins of the ureteral stricture. The ureteroscopic method is also useful to confirm a patent and watertight anastomosis. However, in patients with near-complete or complete ureteral lumen obliteration, the ureteroscopy cannot access the proximal ureter, and so the authors chose the method of injecting indocyanine green (ICG) into the ureteral lumen to identify the precise ureteral stricture location under NIRF. Alternatively, ICG was intravenously injected to assess the ureteral perfusion. Under subsequent NIRF, the well-perfused ureter fluoresced green, while the strictured ureter fluoresced poorly or did not fluoresce [26, 27].

### 3.2. Is a Buccal or Lingual Mucosa Graft Preferred?

BMG harvesting has been demonstrated to be associated with some long-term donor site morbidities, such as perioral numbness, persistent difficulty with mouth opening, and latent parotid duct injury [28, 29]. Innovatively, Li et al. reported their initial experience with laparoscopic onlay ureteroplasty with a lingual mucosal graft (LMG) for repairing proximal ureteral stricture in one patient and obtained excellent outcomes at the 9-month follow-up [30]. The LMG was harvested from the ventrolateral surface of the tongue, where the mucosa has no particular functional features but has histological features identical to the rest of the oral mucosa. The submucosal muscle and adipose tissue of the LMG were removed to create a thin patch graft. Finally, the graft was incorporated into the strictured ureter as a ventral onlay and then wrapped in the omentum [30]. If a longer graft is needed, the harvesting procedure can be extended to the opposite side across the tip of the tongue in continuity. The bilateral ventrolateral aspects of the tongue can provide an LMG reaching up to 11-17 cm in length and 2 cm in width in an adult, but this depends on the scale of the tongue [31].

There is great controversy as to whether the buccal mucosa or lingual mucosa provides a better graft. Because there is limited experience of OMG onlay ureteroplasty, we obtained comparative information from studies of OMG urethroplasty. Lumen et al. had demonstrated that LMG provides outcomes equivalent to BMG urethroplasty for anterior urethral stricture but with lower donor site morbidity [32]. There is more bleeding when harvesting an LMG because the tongue is more vascular than the cheek. However, LMG harvesting is technically easier than graft harvesting from the inner check because the tongue can be pulled out of the mouth [33]. Moreover, morbidities after LMG harvesting, such as difficulty in mouth opening and persistent numbness of the donor site, were significantly fewer than those after BMG harvesting [32]. As the tongue is more involved in speaking, gustation, and movement than the cheek, these related complications would be more likely to occur with LMG harvesting. In a recent study by Xu et al., 34.6% patients (28/81) experienced mild to moderate difficulty with fine motor movements of the tongue, in which 27.2% (22) had associated numbness over the donor site, 12.3% (10) experienced parageusia, and 13.6% (11) reported slurred speech 6 months postoperatively [31]. All patients reported a restriction of tongue protrusion 24 hours postoperatively, and most cases reported pain or discomfort at the donor site within the first 3 days postoperatively. The patients had a higher occurrence rate of donor site complications with LMG longer than 12 cm or bilateral harvesting [31]. Similar to the BMG, nonclosure of LMG donor site might also help in reducing the restriction to tongue movement when a long or bilateral LMG harvesting is needed. Fortunately, donor site complications have been demonstrated to be confined to the first year after operation [31, 34].

Patients who smoke or those who chew betel quid (leaf or nut) usually have poor oral hygiene and unhealthy buccal mucosae [35, 36]. Therefore, the LMG is an excellent alternative, as the lingual mucosa scarcely comes in contact with the quid. In patients with relatively long strictures, a BMG is not enough for graft reconstruction, and LMG in combination with BMG can be considered [37]. However, all these techniques are experimental and the better graft is to be determined.

### 3.3. Patched versus Tubularized OMG

Tubularized BMG interposition for the management of complex ureteral stricture in humans was initially reported by Naude and was applied to only one patient with a traumatic loss of 4 cm of the middle ureter [19]. Good patency and drainage were observed on the retrograde pyelography, but an abnormal appearance was observed on the radiographs 3 months after the surgery. Subsequently, Badawy et al. presented the first series (5 cases) of buccal mucosa tubularized grafts for proximal and middle ureteral reconstruction in 2010 [38]. The ureteral strictures of a 4.4 cm average length resulted from chronic inflammatory conditions or iatrogenic procedures, and the clinical and radiological results of tubular BMG were encouraging at a mean follow-up of 24 months. In this series, the vascularized ureteral adventitia was preserved after the excision of the diseased ureter in some cases, and the reconstructed ureter was wrapped with a pedicled piece of omentum in all cases to maintain a suitable blood supply. However, whether the ureteral adventitia bed is sufficient to allow a successful graft take is obscure [38]. Recently, Fahmy et al. reported a case involving the whole circumference substitution of a 6 cm proximal ureter using tubularized BMG, and they sutured the two ends of the ureter with the BMG in a spiral shape fashion to minimize the possibility of anastomotic stricture [39]. The patient achieved good clinical and radiological outcomes 12 months postoperatively. However, a longer follow-up is still required to confirm the feasibility of this procedure.

Some urologists reported that tubularized grafts might be associated with a higher rate of restricture or fibrosis formation than onlay grafts for the ureteral reconstruction of the same length [40, 41]. Failure of the tubularized graft may be mainly attributed to the poor blood supply, resulting in an inadequate graft “take.” However, if there is not enough “ureteral plate” for OMG onlay repair, tubularized OMGs with wrapped omentum could be alternative for ureteral reconstruction. Based on the current experience, OMG onlay ureteroplasty may obtain better outcomes than tubularized graft ureteroplasty, but more studies with larger samples and comparative analyses are of considerable necessity.

## 4. Intestinal Flap Ureteroplasty

One of the typical approaches for long-segment ureteral reconstruction is to incorporate a pedicled bowel segment, especially an ileal ureter, to bridge the gap between the kidney and the bladder. Although satisfactory outcomes are obtained in the initial follow-up, there are some noticeable long-term complications of simple intestinal ureteral substitution, including recurrent urinary tract infections, metabolic disturbance, mucous production, and a potential deterioration of renal function [42]. However, ureteral reconstruction using an intestinal onlay flap seems to be free of the above complications. Several reports have demonstrated the success of ileal or appendiceal flap ureteroplasty (Table 2). And the reported length of ileal or appendiceal flap ureteroplasty ranged from 1 to 8 cm with success rates of 75%-100%.

Originally, Gomez-Avraham et al. reported the application of ileal flaps to reconstruct complex upper or midureteral strictures in 4 patients [43]. The patients with severe stricture of the ureter were resistant to prior attempts at traditional procedures but recovered adequate renal and ureteral function after ileal flap ureteroplasty. During the operative procedure, the strictured segment of the ureter was longitudinally incised, and a “V”-shaped incision was made in the upper and lower margins of the dissected ureter to form the ureteral bed. One-third of the circumference of the antimesenteric border of the pedicled ileum was preserved as the ileal patch and then anastomosed to the ureteral bed with the mucosa sutured in the lumen [43]. In addition, Antonio et al. attempted to introduce reversed ileal seromuscular patch for ureteral reconstruction but received unsatisfactory results [44]. Intestinal flap ureteroplasty has many significant advantages over simple ileal ureter replacement or appendiceal interposition, including minimized mucous production and the elimination of metabolic complications. However, it is uncertain whether patch-tissue reconstructed ureters would preserve the characteristic of peristalsis similar to that of the native ureter or simply similar to that of simple intestinal replacement after complete surgical excision. In addition, it remains to be determined whether intestinal flap ureteroplasty shows clinical and functional benefits over the Yang-Monti technique. We presume that ureteroplasty with intestinal flap is more suitable for the repair of ureteral strictures with relatively short lengths, but the length threshold is open to debate. When the available intestine is not sufficient, such as with an adhesive ileus or short bowel syndrome, an onlay repair technique should be performed with caution.

**Figure 1:**
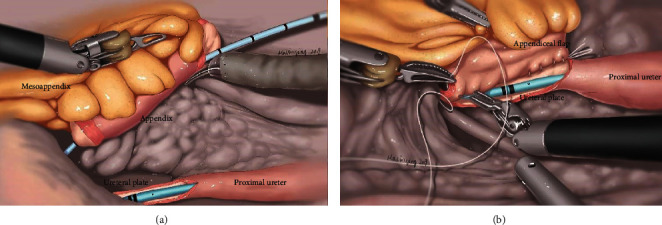
Ureteroplasty using the onlay repair technique (taking robotic appendiceal onlay flap ureteroplasty as an example) [66]. (a) Partial circumference of the strictured ureter was removed to create a ureteral plate; thereafter, the appendix was excised from the colon retaining blood vessel pedicle; then, it was incised longitudinally on its antimesenteric border, forming an appendiceal flap. (b) The appendiceal flap was mobilized to finish the anastomosis with the ureteral plate.

Reggio et al. reported the first laparoscopic case of using reconfigured appendix as an onlay flap for ureteroplasty. The radiological analysis showed good patency of the ureter, and the patient denied any passage of mucous 8 months postoperatively [45]. Subsequently, the authors provided long-term follow-up of this patient and their related experience with five additional cases in another report [46]. The mean stricture length was 2.5 cm, and the mean (range) follow-up was 16.3 (3.8-30.4) months. The objective success rate was 100% with improved hydronephrosis and normal urinary drainage. The subjective success rate was 66%, with two patients developing recurrent pain due to fibrosis of the appendiceal flap. Overall, appendiceal onlay ureteroplasty is a viable approach for the repair of complex proximal and midureteral strictures, decreasing the potential morbidity and preserving the benefits of appendiceal interposition [46]. Recently, our group has applied the appendiceal flap ureteroplasty techniques outlined above to a minimally invasive, robot-assisted approach. As shown in Figure 1, firstly, partial circumference of the strictured ureter was removed. Subsequently, the appendix was excised from the colon, and then, it was opened longitudinally on its antimesenteric border, forming a pedicled appendiceal flap. Finally, the appendiceal flap was mobilized to complete the anastomosis with the ureter.

## 5. Urogenital Grafts Ureteroplasty

The urothelium of the renal pelvis, ureter, and bladder has similar characteristics. Ureteral reconstruction with bladder mucosa grafts or renal pelvis wall grafts seems to be quite reasonable. Macauley and Frohbose reported the successful surgical reconstruction of ureteropelvic junction stenosis using free renal pelvis wall patch grafts in 9 patients [47]. The incision started at the wall of the renal pelvis and extended through the stenosed ureteropelvic junction into the ureter of the normal lumen. The patch grafts were obtained from the posterior aspect of the wall of the renal pelvis and were then immediately sutured in place at the repair sites. All patients recovered adequate renal function and maintained good patency and urinary drainage without recurrent stenosis. Moreover, the authors emphasized that when the wall of the renal pelvis is severely thickened or inflamed or when the ureteropelvic junction is badly scarred and avascular, this operation is inappropriate [47].

The bladder mucosa is an attractive graft for ureteral reconstruction, as it is resistant to urine exposure and abundant in blood supply and has great distensibility [48]. Urban et al. reported the use of bladder mucosa grafts that were harvested under ureteroscopy, and they were subsequently placed into the incised ureteral bed to reconstruct ureteral strictures with lengths of 1.5 to 8 cm in 6 patients [49]. Five patients (83.3%) had a patent ureter and relief of symptoms at a long-term (>22 months) follow-up. Moreover, Zou et al. demonstrated that it was feasible to repair ureteral defects and strictures using bladder mucosa grafts without a vessel pedicle in animal experiments [50]. Postoperative examinations identified the patency of neoureters and the survival of bladder mucosa grafts. Furthermore, the authors supposed that the blood supply from periureteral connective tissues was sufficient to nourish the grafts, and the bladder has sufficient tissues for the required length of the neoureter regardless of the bladder capacity [50]. Kuzaka et al. evaluated the blood supply of the ureters reconstructed with free bladder mucosa grafts by microangiography, and they found that the mucosa of the reconstructed portion of the ureters was completely regenerated, but there was an absence of revascularization and the regeneration of the muscular coat, which caused dense scarring or stricture of the ureters and massive periureteral fibrosis [51]. In addition, other problems may also arise, such as hypertrophy, prolapse, and a granulomatous reaction [52, 53]. Therefore, more investigations are critically needed to evaluate the long-term functional efficacy of this technique.

Penile/preputial skin is a popularized onlay graft for urethral reconstruction, with the advantage of being devoid of hair and being fat, flexible and easy to harvest, and acceptable donor site morbidity [54]. The use of penile/preputial skin patch grafts for the reconstruction of ureteral strictures has also been reported. Onal et al. presented the initial case report of preputial onlay graft ureteroplasty for a complex proximal ureteral stricture 5 cm in length [55]. The graft was harvested from the ventral side of the penis, and then, the submucosal muscle and adipose tissue were excised to create an ultrathin patch graft. The reconstructed ureter was covered with an omental wrap to augment vascularity. The renal function and ureteral urine drainage of this patient were normal 9 months after the operation [55]. However, high recurrence of contracture and restenosis is given as the disadvantage of penile/preputial skin graft in long-term follow-up [55]. And larger-scale investigations with longer follow-ups are needed to evaluate and optimize this technique. In addition, more reports with respect to autologous tissue patch graft ureteroplasty are presented in Table 2.

## 6. Tissue Engineering and Regeneration Based Ureteroplasty

When surgical repairs, such as bowel substitution, renal autotransplantation, or even BMG ureteroplasty, is not available or does not succeed, a nephroureterectomy procedure is inevitable. Alternatively, ureteral tissue engineering is an emerging field for developing an optimal material for ureteral reconstruction and avoiding the problems of autologous tissue grafts, including donor site morbidity and time-consuming harvesting [56]. So-called scaffolds are the building blocks to promote tissue regeneration, which could be produced from decellularized native tissue. Scaffolds can be further categorized on the basis of whether they are directly implanted, seeded with cells prior to implantation, or preimplanted before functional implantation [57]. The small-intestinal submucosa (SIS), a heterologous, biocompatible, nonimmunogenic collagen matrix originating from the porcine intestinal submucosal layer, is a resorbable biological scaffold commonly used through direct implantation. The SIS has been demonstrated to facilitate the successful regeneration of host tissues from bench to bedside, including those used for ureteral reconstruction the in preclinical studies (as shown in Table 3).

Initially, Liatsikos et al. evaluated the effectiveness of SIS patch grafts for left proximal ureteral reconstruction in 6 female pigs, and the right ureters served as the control [58]. Histological results revealed a regenerated epithelium and neovascularization along SIS-reconstructed segments, and retrograde pyelography confirmed patency in the reconstructed ureters 7 weeks after the initial procedure, while the right ureters showed no evidence of epithelial regeneration [58]. Smith et al. also demonstrated that the patch graft technique using the SIS successfully induced ureteral regeneration. After 9 weeks, the SIS patch graft was replaced by regenerative ureteral tissue, including transitional epithelium with focal intestinal metaplasia, a submucosa, and normal ureteral musculature, significantly similar to normal porcine ureters [59]. Liatsikos et al. speculated that urothelial regeneration originated from the “ureteral plate” with remaining one-third of the diameter, while Smith et al. believed that the regeneration of the urothelial lining depends on the adjacent urothelium from the upper and lower ureteral ends of the anastomosis [58, 59]. As shown in Table 3, SIS onlay grafts resulted in apparently better functional and regeneration outcomes than tubularized SIS grafts in preclinical studies. The addition of autologous cells to scaffolds might enhance tissue regeneration, but a cell-seeded SIS does not improve the functional outcome when compared to an implanted nonseeded SIS.

## 7. Conclusion

Long-segment stricture of the proximal and midureter is often a treatment dilemma for most urologists. Ureteroplasty using autologous patch grafts is an alternative surgical technique, namely, an onlay ureteroplasty procedure with or without an augmented anastomotic technique (Figure 1). The previously reported autologous grafts for the repair of ureteral strictures include the oral mucosae (buccal and lingual mucosae), ileal and appendix mucosae, renal pelvis wall, bladder mucosae, penile/preputial skin, and vein. BMGs have gained wide acceptance as a graft choice for ureteroplasty, but an optimal onlay graft is yet to be further developed. As shown in Table 4, each graft type has unique superiorities and potential problems. Tissue engineering and regeneration-based ureteroplasty may provide alternative solutions for ureteral reconstruction when autologous tissues are not accessible or are insufficient. Ureteral tissue engineering can develop optimal materials, and this strategy may be adopted as a standard technique for the management of ureteral strictures in the future. As there are limited data on onlay graft ureteroplasty, more studies are needed to standardize the success and efficacy of this procedure.

## Figures and Tables

**Table 1:** Clinical series reports of ureteroplasty using oral mucosa grafts.

Authors and year	Patients (*n*)	Type of the graft	Length of repair (cm)	Follow-up (months)	Donor site complications	Recipient site complications	Success rate^c^ (%)
Naude (1999) [18]	4	BMG (open onlay 3; tube 1)	4^a^	3-72	Not determined	None	100
Shailesh et al. (2003) [60]	5	BMG (open onlay)	5.5-9.0	18-42	Not determined	None	100
Kroepfl et al. (2009) [23]	7	BMG (open onlay)	3-11	10-85	Not determined	Restenosis in 2 (one occurred 39 months later; another 17 months)	71.4 (5/7)
Badawy et al. (2010) [38]	5	BMG (open onlay)	3.5-5.0	14-39	Not determined	None	100
Pandey et al. (2014) [61]	3	BMG (open onlay)	4-6	26-50	Not determined	None	100
Zhao et al. (2015) [24]	4	BMG (robotic onlay, anterior in 2, posterior in 2)	1.5-6.0	10.7-18.6	Not determined	None	100
Li et al. (2016) [30]	1^b^	LMG (laparoscopic onlay)	4	9	None	None	100
Tsaturyan et al. (2016) [62]	5	BMG (open onlay)	2.5-5.0	26-52	Not determined	None	100
Lee et al. (2017) [63]	12	BMG (robotic onlay)	2-5	4-30	Not determined	Stricture recurrence in 2	83.3 (10/12)
Ahn et al. (2017) [64]	3	BMG (robotic onlay)	2.5-6	5-26	None	None	100
Zhao et al. (2018) [25]	19	BMG (robotic onlay, ventrally in 15, dorsally in 4)	2-8	13-44	Not determined	Restenosis in 2 (one occurred 1 year later, another 6 weeks)	89.4 (17/19)
Hefermehl et al. (2020) [41]	4	BMG (open onlay)	3-5	12-14	Difficulties to whistle in 1	None	100

^a^Authors only mentioned the reconstructed length of tube graft; ^b^ first report of ureteroplasty using lingual mucosa graft; ^c^success was defined as patent drainage and free of stricture recurrence.

**Table 2:** Clinical series reports of onlay ureteroplasty using autologous grafts (excepting OMG).

Authors and year	Patients (*n*)	Type of the graft	Length of repair (cm)	Follow-up (months)	Donor site complications	Recipient site complications	Success rate^b^ (%)
Gomez-avraham et al. (1994) [43]	4	Ileal flap (open onlay)	2-6	6-18	Not determined	None	100
Ordorica et al. (2014) [65]	9	Ileal graft in 7, appendiceal graft in 2 (open onlay)	5-8	12-78	None	Ureteral fistula in 1	88.9 (8/9)
Duty et al. (2015) [46]	6	Appendiceal flap (laparoscopic onlay)	1-6	3.8-30.4	Not determined	None	100
Wang et al. (2020) [66]	9	Appendiceal flap (laparoscopic onlay in 5, robotic onlay in 4)	3-4.5	4-10	None	None	100
Macauley and Frohbose (1970) [47]	9	Renal pelvis wall graft (onlay)	Not determined	12	Delayed emptying of the renal pelvis in 3	None	75 (9/12)
Urban et al. (1994) [49]	6	Bladder urothelial graft	1.5-8	15-54	None	Stricture recurrence in 2 (occurred 15 and 12 months later)	66.7 (4/6)
Onal et al. (2018) [55]	1^a^	Preputial skin patch graft	5	12	None	None	100
Pompeius et al. (1977) [67]	4	Vein patch graft	2-3	6-120	Not determined	None	100

^a^First report of ureteroplasty using preputial skin patch graft. ^b^Success was defined as patent drainage and free of stricture recurrence and fistula.

**Table 3:** Preclinical studies regarding tissue engineering-based ureteroplasty.

Authors and year	Animal model, *n*	Type of scaffold	Length of repair (cm)	Follow-up (weeks)	Functional outcomes of recipient site	Regeneration outcomes of recipient site
Liatsikos et al. (2001) [58]	Pig (F), 6	SIS (onlay)	7	7	Good patency and anastomoses	U, S, N
Smith et al. (2002) [59]	Pig (F), 9	SIS (onlay)	2	9	Good patency	U (with focal intestinal metaplasia), S, N
Greca et al. (2004) [68]	Pig, 10	SIS (onlay)	2	5.7	Good patency in 7, fistula in 1, restenosis in 2	U and N (in 100% cases), S (in 87.5%)
Duchene et al. (2004) [69]	Pig, 12	SIS (onlay in 5, tube in 7)	2	6 or 9	Patent in patch group, complete obstruction in tube group	Patch group: U, S, Fi, and I (mild); tube group: U, S (partial), F (dense)
El-Hakim et al. (2005) [70]	Pig (F), 8	SIS (tube)+UCs/SMCs	5	6	Contraction and stenosis	U, S, Fi (dense)
de Jonge et al. (2018) [71]	Pig (F), 20	Collagen-Vicryl (tube)	5	12.8	Contraction	U (in 32% cases), S (in 50%), N
de Jonge et al. (2018) [72]	Goat (F), 12	Collagen-Vicryl (tube)+subcutis	1.5-3.5	12.8	Patent in 8, urine leakage in 2, stenosis in 2	U, N, S (limited to the anastomosis sites), I (mild)

F: female; SIS: small intestine submucosa; UCs: urothelial cells; SMCs” smooth muscle cells; U: urothelial regeneration; S: smooth muscle ingrowth; N: neovascularization; Fi: fibrosis; I: inflammation.

**Table 4:** Characteristics of the reported autologous patch grafts for ureteroplasty.

Drafts	Advantages	Disadvantages
Buccal mucosa graft	Easy to harvest and handle, tolerance to wet environment, good graft “take”	Adversely affected by smoking and betel quid chewing; donor site morbidity such as persistent difficulty with mouth opening and latent parotid duct injury
Lingual mucosa graft	Easy to harvest, tolerance to wet environment, obtainable long-segment graft harvesting, good graft “take”, acceptable donor site morbidity	Thin and relatively hard to handle; potential donor site morbidity such as difficulty with fine motor of the tongue and slurred speech
Ileal mucosa flap	Much less intestine needed than simple replacement, much diminished risk of metabolic derangement and mucous production	More difficult to harvest and handle; infeasible when ileal inflammation or other diseases occur; potential donor site complications including intestinal anastomotic infection or leak, anastomotic hemorrhage or stenosis, and adhesive or paralytic intestinal obstruction
Appendiceal flap	Much less intestine needed than simple replacement, much diminished risk of metabolic derangement and mucous production, maintaining its own blood supply from the appendicular artery	Infeasible when appendiceal inflammation or calculus occurs; potential donor site complications including intestinal anastomotic leak
Renal pelvis wall graft	Grafts close to reconstructed field, similar to the tissue characteristics of the ureter	Limited data; limited area of graft harvesting; preferably to reconstruct the obstruction of ureteropelvic junction; large graft harvesting may damage the neuromuscular mechanism of renal pelvis
Bladder mucosa graft	Obtainable long-segment graft harvesting, tolerance to urine corrosion, and minimized stone formation	Donor site morbidity; complications including stricture, hypertrophy, prolapse of the reconstructed site
Penile/preputial skin graft	Devoid of hair and fat, easy to harvest and handle	Limited data; high recurrence of contracture and restenosis
Vein patch graft	Easier to harvest and handle; usually available nearby the spermatic or ovarian vein	Limited data; confined to repairing short strictures; complications including fibrosis and restenosis of the reconstructed ureters
